# Neonatal iron distribution and infection susceptibility in full term, preterm and low birthweight babies in urban Gambia: study protocol for an observational study.

**DOI:** 10.12688/gatesopenres.12963.2

**Published:** 2019-10-15

**Authors:** James H. Cross, Ousman Jarjou, Nuredin Ibrahim Mohammed, Andrew M. Prentice, Carla Cerami

**Affiliations:** 1 MRC Unit The Gambia at the London School of Hygiene & Tropical Medicine, Fajara, The Gambia

**Keywords:** Nutritional Immunity, Host-Pathogen Interaction, Hepcidin, Neonates, Hypoferremia, Transferrin, The Gambia, Sub-Saharan Africa

## Abstract

**Background: **Neonatal infection is the third largest cause of death in children under five worldwide.  Nutritional immunity is the process by which the host innate immune system limits nutrient availability to invading organisms. Iron is an essential micronutrient for both microbial pathogens and their mammalian hosts. Changes in iron availability and distribution have significant effects on pathogen virulence and on the immune response to infection. Our previously published data shows that, during the first 24 hours of life, full-term neonates have reduced overall serum iron. Transferrin saturation decreases rapidly from 45% in cord blood to ~20% by six hours post-delivery.

**Methods: **To study neonatal nutritional immunity and its role in neonatal susceptibility to infection, we will conduct an observational study on 300 full-term normal birth weight (FTB+NBW), 50 preterm normal birth weight (PTB+NBW), 50 preterm low birth weight (PTB+LBW) and 50 full-term low birth weight (FTB+LBW), vaginally-delivered neonates born at Kanifing General Hospital, The Gambia. We will characterize and quantify iron-related nutritional immunity during the early neonatal period and use *ex vivo* sentinel bacterial growth assays to assess how differences in serum iron affect bacterial growth. Blood samples will be collected from the umbilical cord (arterial and venous) and at serial time points from the neonates over the first week of life.

**Discussion: **Currently, little is known about nutritional immunity in neonates. In this study, we will increase understanding of how nutritional immunity may protect neonates from infection during the first critical days of life by limiting the pathogenicity and virulence of neonatal sepsis causing organisms by reducing the availability of iron. Additionally, we will investigate the hypothesis that this protective mechanism may not be activated in preterm and low birth weight neonates, potentially putting these babies at an enhanced risk of neonatal infection.

**Trial registration: **clinicaltrials.gov ( NCT03353051) 27/11/2017

## Abbreviations

PTB = Preterm birth

FTB = Fullterm birth

LBW = Low birthweight

NBW = Normal birthweight

WHO = World Health Organisation

TSAT = Transferrin saturation

MDG = Millennium development goal

GBS = Group B *Streptococci*

EONS = Early onset neonatal sepsis

YICSS = Young Infants Clinical Signs Studies

KGH = Kanifing General Hospital

MRCG = Medical Research Council Unit The Gambia at LSHTM

KMC = Kanifing Municipal Council

UIBC = Unbound iron-binding capacity

IL6 = Interleukin 6

IL22 = Interleukin 22

sTfR = Soluble transferrin receptor

CRP = C-reactive protein

AGP = Alpha 1-acid glycoprotein

HIV = Human immunodeficiency virus

TB = Tuberculosis

EDTA = Ethylenediaminetetraacetic acid

V1 = Venous bleed 1

V2 = Venous bleed 2

IM = Intramuscular

ELISA = Enzyme-linked immunosorbent assay

ID = Identification

CRF = Case report form

eCRF = Electronic case report form

ANOVA = Analysis of variance

IDE = Integrated development environment

CyTOF = Cytometry by time of flight

STAT3 = Signal transducer and activator of transcription 3

TLR = Toll-like receptor

SOP = Standard operating procedure

OD = Optical density

WBC = White blood cell

LPS = Lipopolysaccharide

## Introduction

### Neonatal infections – challenges in low-income settings

Neonatal infection is the third largest cause of death in children under-five worldwide and is an ongoing major global public health challenge (Sustainable Development Goal 3.2) ^1^. Between 1990 and 2016 maternal and under-five child mortality has decreased by half ^2^. However, the proportion of neonatal deaths among under-five deaths increased from 37% (1990) to 44% (2013) ^3, 4^. Today, approximately 2.8 million children die annually during the neonatal period – the first 28 days of life. Of these, 73% die within the first six days of life ^3^. An increasing proportion of child deaths are in sub-Saharan Africa ^5^, with 60–80% of newborn deaths occurring in low birthweight (LBW) neonates (<2500g at birth) ^6^. 95% of all LBW neonates are born in low-income countries ^7^. However, the situation is likely to be worse than documented, as neonatal deaths in developing countries are commonly under reported and the records commonly contain errors ^8, 9^. It is estimated that about one third of deaths in the first month of life, are caused by infections including bacterial sepsis, meningitis, pneumonia, neonatal tetanus, and diarrhoea ^10^.

Evidence is lacking on the aetiology of neonatal infections in developing countries, especially from community settings ^11, 12^. However, the limited data suggests that *Klebsiella* species, *Escherichia coli,* and *Staphylococcus aureus* are common causes of early onset sepsis (EONS) ^13– 15^. The available antibiotic susceptibility data suggests that pathogens associated with neonatal sepsis in developing countries are often resistant to WHO-recommended empiric antibiotics ^13, 16^. Antibiotic resistance has emerged with potency over the last few decades due to a multitude of complex reasons. Antibiotic overuse, inappropriate prescribing, inadequate diagnostics, extensive agricultural use, availability of few new antibiotics, and the ease of transportation of resistant bacteria are among the factors contributing to the rise. Equally, bacteria have the ability to rapidly mutate (with or without drug selection pressure) and horizontally transfer genetic material between species (i.e. non-human pathogens) of bacteria ^17, 18^. Neonates are particularly at risk from antibiotic resistant organisms because they generally succumb before alternative antibiotic regimes can be tried.

Diagnosis of neonatal sepsis with high specificity remains challenging in developing countries. A widely used tool developed by the World Health Organization Young Infants Clinical Signs Studies (YICSS), which includes seven clinical signs to aid diagnosis, has only a 85% sensitivity and 75% specificity for severe bacterial infection during the first week of life ^19^. Microbiological identification of a pathogen isolated from blood cultures often has higher specificity, but microbiological laboratory facilities are frequently lacking in low-income settings ^19^. With this all in mind, there is an immediate need to improve our understanding of neonatal blood-borne infections and develop novel therapies that could enhance immunological protection possibly via boosting innate immune mechanisms.

### Nutritional immunity

Iron is critical for the human host and most pathogens. Iron is one of the most important factors in the host-pathogen battle for resources. Bacteria and other pathogens have evolved a wide variety of mechanisms to acquire iron from the nutrient rich host (e.g. siderophores and iron specific channels) ^20^ to aid growth and virulence, with a number of iron acquisition genes concentrated on high pathogenicity islands ^21^.

Nutritional immunity describes the normal physiological innate processes used by the host to combat infection by limiting nutrient availability. Key among these processes is the ability to rapidly decrease the circulating concentration of iron (and other transition metals) in response to an infection ^22^. The hypoferremia of inflammation is mediated by the hormone, hepcidin. Research completed in 2000–2001 by three independent research groups led to the discovery of the hepcidin hormone, and the important function it plays in many aspects of iron metabolism ^23– 25^. Hepcidin is now understood to be the master regulator of iron homeostasis. Unlike any other micronutrient, iron is regulated by a hormone that responds to both infection and nutritional status. The host inflammatory mediators, IL6 ^26^, IL22 ^27^ and Type 1 interferon ^28^, have been found to increase transcription of hepcidin through several Toll-like receptor (TLR) ligands ^29^ and STAT3 signalling ^30, 31^ resulting in decreased systemic iron concentrations in the circulation. This multifaceted mechanism limits nutrient availability to extracellular invading microorganisms ^32^. The system is well documented in mouse models ^33– 35^, but less so in human studies. However, it is clear that humans with excessive levels of serum iron (e.g. due to hemochromatosis) are predisposed to infection with iron-dependent species of bacteria ^36, 37^.

### Neonatal hypoferremia

Although iron metabolism in adults and older children is well studied, the kinetics of iron handling in the early neonatal period, a time of intense physiological change, are poorly understood ^38^. Childbirth results in a neonate moving from a semi-allogeneic, protected and nearly sterile environment to one that is abundant in a diverse array of microbes. The delivery process is the initial focal point for the mass bacterial colonisation of the skin and gastrointestinal tract of the neonate ^39, 40^. Neonates are known to have very low levels of immunological memory and possess an immature immune system ^41^. Post-natal iron metabolism in neonates is controlled by an array of different signals, such as hypoxia, erythropoietic drive, maternal and foetal iron stores ^42^. A number of studies have investigated serum iron, transferrin saturation (TSAT), ferritin and haemoglobin levels at the time of birth using cord blood as a proxy for early neonatal blood ^43, 44^. A recent prospective study showed neonates born preterm compared to early-term had higher serum iron concentrations in umbilical blood, which was inversely correlated with levels of serum hepcidin ^45^. A similar study has also shown that small-for-gestational-age neonates and neonates born by elective caesarean have lower levels of hepcidin ^44^. Previous work has shown that serum iron and TSAT decreases between birth and the first 6–12 hours post-partum in full term, healthy vaginally delivered newborns ^46, 47^.

The study described here will shed light on the effects of prematurity and birthweight on body iron distribution immediately after birth and during the first week of life. Free ferric and ferrous iron (i.e. transferrin bound iron), haem-based iron molecules and their chaperone proteins (haem-hemopexin and haemoglobin-haptoglobin) will also be investigated.

### Study objectives

The primary study objective is to characterize in detail how full term, preterm and low birthweight neonates modulate serum iron in the first 24 hours of life. We hypothesize that premature and/or low birthweight babies have a defect in their ability to sequester iron at 6–24 hours after birth in comparison to full term neonates with normal birthweight.

The secondary objectives are:

I. Characterise how iron metabolism, handling and recycling differs between full term, preterm and low birthweight neonates at birth and during the first 24 hours of life.
II. Describe iron metabolism, handling and recycling in full term neonates at birth and during the first 7 days of life.
III. Determine if sera from preterm and low birthweight neonates supports a greater level of *ex-vivo* growth of microorganisms that are common causes of neonatal sepsis in Africa and The Gambia ( *Staphylococcus aureus*, *Klebsiella pneumoniae*, *Escherichia coli*, Group B *Streptococcus*, *Streptococcus pneumoniae* and *Salmonella enterica* serovar Typhimurium ( *S.* Typhimurium hereafter)) in comparison to sera from full term, normal birthweight neonates.
IV. Characterize frequencies and functionality of neutrophils, monocytes, dendritic cells, NK cells, B cells and T cells (D8 and CD4) in cord blood from full term, premature and low birthweight neonates.

## Protocol

### Study site

Study participants will be recruited from Kanifing General Hospital (formally Serrekunda General Hospital), in the Kanifing region of The Gambia, West Africa. Serrekunda is a large town, forming a peri-urban area with a population of around 340,000, and is 13km to the southwest of the capital, Banjul. Serrekunda was originally made up of nine villages that have merged into a sprawling urban area. Annually, Kanifing General Hospital (KGH) provides antenatal care to 500–700 pregnant mothers. Mothers receiving antenatal care at other local healthcare facilities increase the total number of births at the hospital to 3500–4500 per year. The percentage of these that are live, low birthweight neonates (<2.5kg) is approximately 10%. Specimen samples will be subjected to primary processing on-site at KGH, followed by transport to Medical Research Council Unit The Gambia at LSHTM (MRCG) for storage and analysis.

### Participants

**Figure 1.**
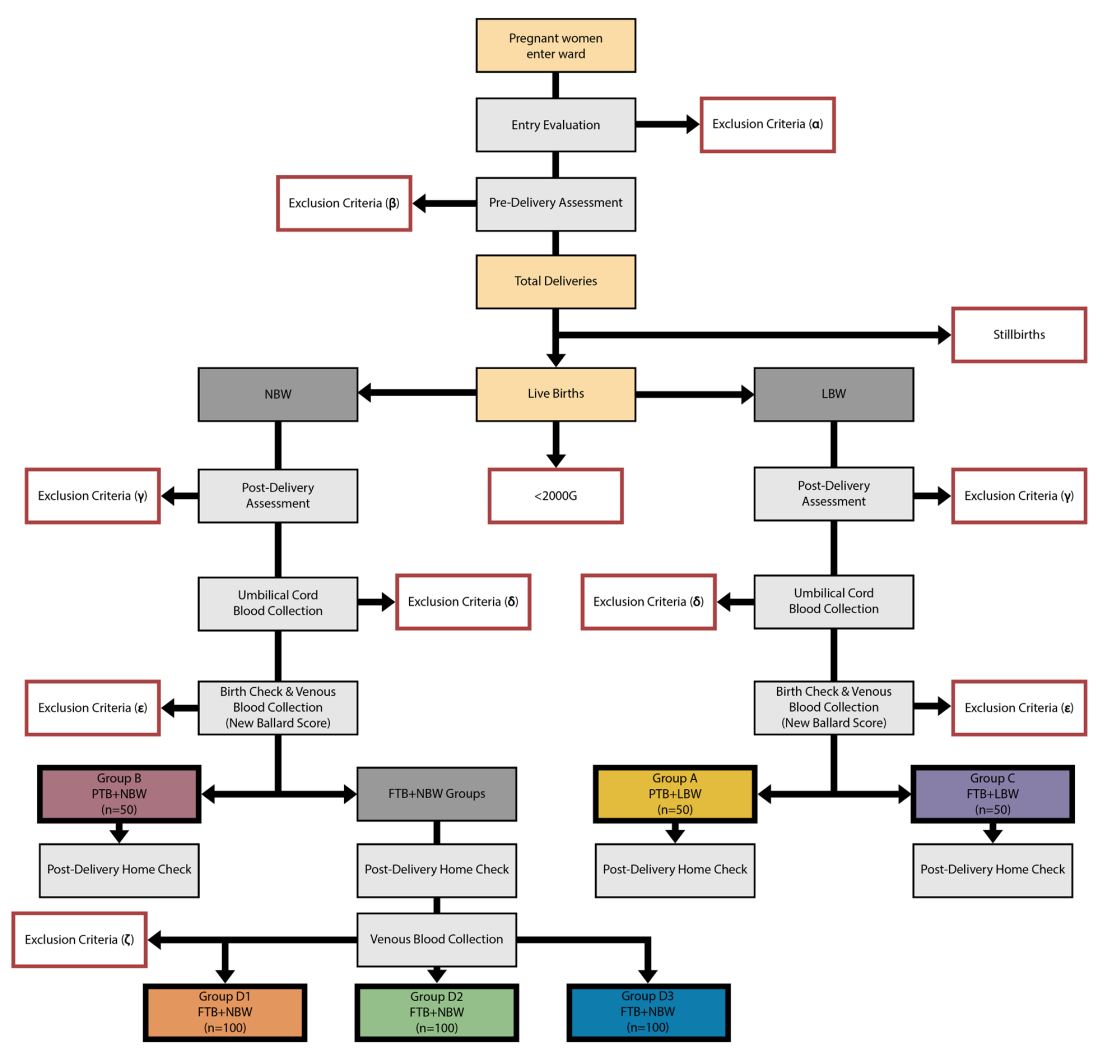
Main study flow chart of all study procedures and exclusion criteria. Group A will contain neonates characterised by preterm birth and low birthweight (PTB+LBW); Group B will contain neonates characterised by preterm birth and normal birthweight (PTB+NBW); Group C will contain neonates characterised by full term birth and low birthweight (FTB+LBW); Groups D1, D2 and D3 will all contain babies characterised by full term birth and normal birthweight (FTB+NBW). In this study, preterm is defined <37 weeks gestation and low birthweight is <2500g. Exclusion criteria ( **α**): Father refused, mother refused, family/escort refused, communication not possible or mother with severe disabilities. Exclusion criteria ( **β**): Antibiotics or antimalarials given before delivery (<24 hours), referred to tertiary level health facility, absconded, known HIV-positive, severe pre-eclampsia, receiving TB treatment, antepartum haemorrhage, recent blood transfusion (within the last month), no foetal heartbeat, mother <18 years, refusal, recruited to another study and emergency caesarean section. Exclusion criteria ( **γ**): Recruited to another study on-site, refusal, blood transfusion given in labour, antibiotics or antimalarials given during labour, neonate requires resuscitation (1 min APGAR), neonatal weight <2000g, neonate born breech, neonate born via vacuum delivery, neonate born caesarean section, foetal stillbirth, macerated stillbirth and major congenital malformations. Exclusion criteria ( **δ**): Failed cord blood collection (serum tubes), failed cord blood collection (EDTA), cord blood processed >6 hours, neonate requires resuscitation (10 min APGAR), absconded and route 2B refusal. Exclusion criteria ( **ε**): Mother birth check refusal, father birth check refusal, family escort birth check refusal, mother <18 years, recruited to another study on-site, antibiotics or antimalarials given to mother before delivery (<24 hours), neonate has had surgery, neonates sibling twin was recruited, neonate given antibiotics (other than tetracycline eye ointment), neonate given iron supplementation, neonatal sickness (tone, activity, feeding, heart rate, respiratory rate, abnormal anterior fontanelle), neonatal temperature (<36.5°C or >37.5°C), major congenital malformations (neonate), New Ballard Score (<32 weeks), failed V1 (serum), failed V1 (EDTA), failed V1 (both EDTA and serum), mother V1 bleed refusal, father V1 bleed refusal, and family/escort V1 bleed refusal. Exclusion criteria ( **ζ**): neonatal sickness (tone, activity, feeding, heart rate, respiratory rate, abnormal anterior fontanelle), neonatal temperature (<36.5°C or >37.5°C), neonate has had surgery, neonate given antibiotics (other than tetracycline eye ointment), neonate given iron supplementation, failed V2 bleed, Mother community/V2 bleed refusal, father community/V2 bleed refusal, and family community/V2 bleed refusal.

In total, 450 healthy newly born neonates will be identified during delivery at the Kanifing General Hospital Maternity Ward ( Figure 1) starting in July 2017. Pregnant mothers must be over the age of eighteen years. After informed consent is obtained, neonates who meet the inclusion criteria will be enrolled into the study. For inclusion in the study, neonates must be healthy, medically stable, greater than 32 weeks gestational age and weigh more than 2000g. To be considered preterm (PTB) the neonates will be < 37 weeks gestational age (assessed by New Ballard Score ^48^) and ≥ 32 weeks gestational age. All neonates with a gestational age ≥37 weeks will be considered full term (FTB). To be considered low birthweight (LBW) the neonates will weigh < 2500g. All neonates weighing ≥ 2500 g will be considered normal birthweight (NBW).

The study groups are:

Group A (PTB+LBW): Neonates who are both preterm and low birthweight.

Group B (PTB+NBW): Neonates who are preterm and normal birthweight.

Group C (FTB+LBW): Neonates who are full term but low birthweight.

Group D (FTB+NBW): Neonates who are full term and normal birthweight.

In addition to the main study, 300 FTB neonates of the 450 neonates will also be included into a sub-study, which aims to describe serum iron markers in full term babies (Group D, FTB+NBW only) over the first week of life.

### Study design

This is a proof-of-concept, observational cohort study (Groups A, B, C and D) with an embedded short prospective cohort study (Group D divided into D1, D2 and D3).

### Entry evaluation

**Figure 2.**
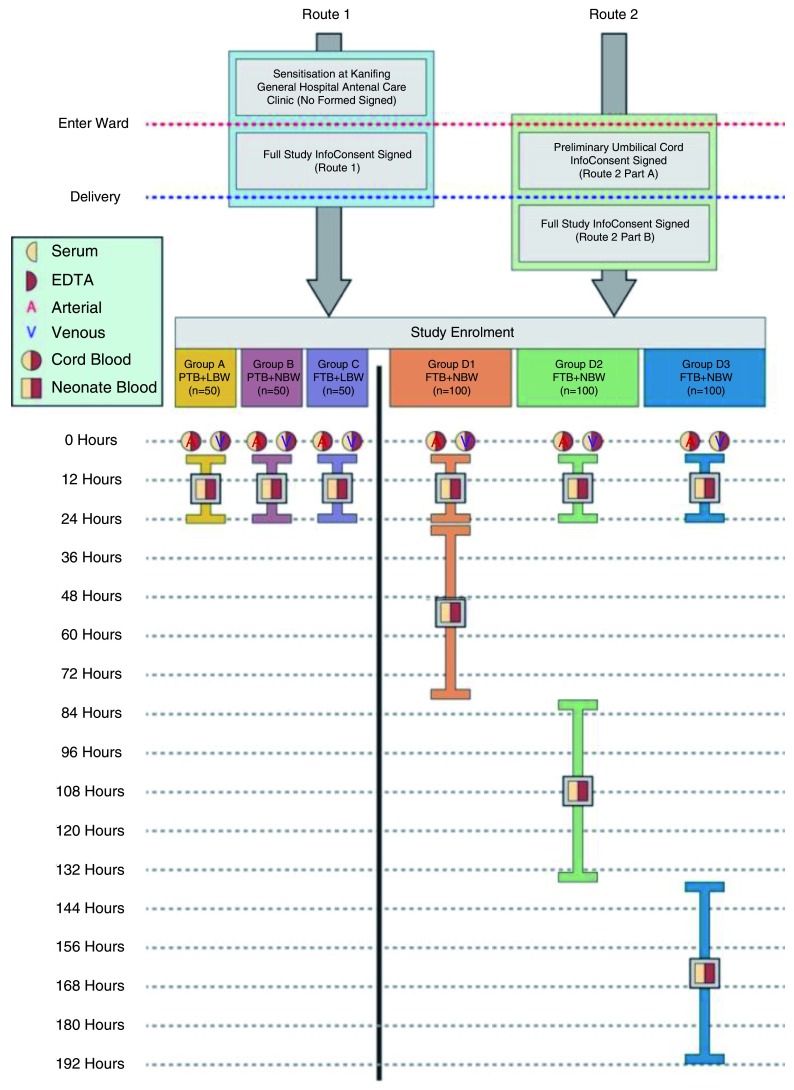
NeoInnate Study enrolment route and blood draw design. Group A contains neonates characterised by preterm birth and low birthweight (PTB+LBW); Group B contains neonates characterised by preterm birth and normal birthweight (PTB+NBW); Group C contains neonates characterised by full term birth and low birthweight (FTB+LBW); Groups D1, D2 and D3 all contain babies characterised by full term birth and normal birthweight (FTB+NBW).

***Consent and enrolment.*** There are two routes into the study enrolment ( Figure 2). Pregnant mothers who are receiving antenatal care on-site at KGH, will be approached at an antenatal visit and voluntarily sensitised to the study requirements and protocol (Route 1). Pregnant women, who are sensitised will not be required at that point to give written or verbal consent. This group will be provided with study information sheets and encouraged to discuss study participation with their family. When the pregnant woman returns to KGH Maternity Ward to deliver (some mothers will choose to deliver at other healthcare facilities), she will be asked to read the full study information sheet (or have it read to her by a study nurse if she is not literate) and provide formal written consent to the study involvement for their neonate (see Extended data ^49– 51^).

Route 2 will provide an alternative route of enrolment for pregnant mothers, that would like their neonate to be part of the study but have been receiving antenatal care at another facility before delivering at KGH maternity ward. In route 2, healthy, pregnant women will enter the KGH ward to deliver and will be approached to provide written formal consent to umbilical cord blood collection and storage only. No testing or laboratory processes will be conducted on their sample, until full study consent is gained post-delivery. The cord blood sample will be stored at 4 ^o^C (within the maternity ward), until the mother’s pain and discomfort subside Mothers will be approached 2-6 hours post-delivery on a one-to-one basis by our research clinician. The research clinician will independently verify if the mother displays normal psychological function and is not under distress before the consenting process begins. At this point, the mother and/or father will be invited to provide written formal consent on full study enrolment. If, at this point, mother and/or father refuse full study consent post-delivery, the previously collected personal information and umbilical cord sample will be safely discarded. Good clinical practise will be followed at all times.

### Pre-delivery screening

In both Route 1 and Route 2 enrolment, mothers must provide written consent before assessment of personal information (antenatal card) and questioning can begin. After consenting, mothers will be asked for their demographic information and their personal contact details. Pregnant mothers will be excluded from the study if they are below the age of 18 years, have no foetal heartbeat detected upon admission, known to be HIV-positive, in receipt of *Mycobacterium tuberculosis* therapy, taken antibiotics in the last 24 hours, had a blood transfusion in the last month, suffering from severe pre-eclampsia or antepartum haemorrhage, or in another research study. Mothers can refuse to be part of the study at any stage of the study protocol. Pregnant women that are referred at this point to a tertiary level healthcare facility, will be excluded from the study.

### Delivery procedures, post-delivery screening and umbilical cord blood collection

***Delivery procedures and screening.*** Study nurses will assist clinical KGH maternity ward staff in the delivery process and collect data via electronic case report form (eCRF) on their designated study tablets. Neonates will be excluded at the delivery stage of the study for the following reasons: major congenital malformations (not including polydactylism), blood transfusions given to mother or neonate, severe birth asphyxia (requiring resuscitation), neonates born via breech, vacuum or via caesarean section, or a birthweight <2000g. After the delivery stage of the study protocol, neonates can be excluded from the study following the detection of infection or illness (information gained from full blood count analysis or review of systems). Neonates will also be removed from the study protocol, if medication is given (not including intramuscular vitamin K, tetracycline eye ointment or any immunisations). All medication that is given to mothers and neonates will be recorded. Mothers will be able to refuse study participation at any stage. Mothers that deliver multiple newborns will only be invited to consent and enrol one of their neonates into the study.

***Umbilical cord blood collection.*** Once the neonate is fully delivered, one-minute delayed cord clamping will be used (following World Health Organisation (WHO) policy ^52^). During the one-minute delay, the one-minute APGAR score will be conducted. If the neonate requires resuscitation, the neonate will be excluded from the study. After the umbilical cord has been removed and cleaned, a trained study nurse will identify the umbilical arteries and the umbilical vein. Blood will be collected from both. The tubes will be placed in the cool box for 1–6 hours before transfer to the study laboratory for primary processing. If the mother is enrolled by route 2, the mother will be asked to provide written consent to full study recruitment before the sample is sent for primary laboratory processing. The time between the collection and the processing of blood samples will be recorded and analysed.

### Hospital assessment and 1 ^st^ venous blood draw

***Hospital health assessment (study recruitment and group allocation).*** At 6–24 hours post-delivery, recruited mothers and their neonates will be invited to a private consultation with the study research clinician. Further demographic data will be collected, along with a photograph of the antenatal card to gather gestational age data (fundal height, last menstrual period and ultrasound), mother’s last haemoglobin level before delivery (dated), known sickle cell status, neonate immunisations, and medication given to the mother (pre, during and post-delivery) and the neonate. A complete review of systems of the mother and neonate plus anthropometric data on the newborn will then be collected. Neuromuscular and physical maturation of each neonate will be assessed using the New Ballard Score ^48^.

Neonates will be excluded if they score less than 32 weeks of gestation. From this assessment, the neonate will be assigned to a specific study group. If the neonate is allocated to the Group D (FTB+NBW) group, the neonate will be allocated to a randomised bleed group (≥24 hours - <80 hours (Group D1); ≥80 hours - <136 hours (Group D2); and ≥136 - <192 hours (Group D3). Failure to meet the inclusion criteria at this stage of the study protocol, will result in exclusion from the study.

According to current practice, at-birth vaccination of neonates at Kanifing General Hospital (The Gambia), does not occur until a later time point in the first week of life due to social and logistical reasons. However, mothers will still be asked whether the neonate has been vaccinated prior to the health assessment before the 1 ^st^ venous bleed takes place. The answers will be recorded and analysed.

***1 ^st^ venous blood draw (all neonates).*** A blood sample will be collected from all neonates that have passed the inclusion criteria in the hospital health assessment. Immediately after the health assessment, a venous blood draw will be performed (6–24 hours post-delivery). PTB and/or LBW neonates will donate 2ml of venous blood. FTB+NBW neonates will donate 3.5ml of venous blood. All samples will reach the laboratory within three hours post collection for primary processing.

### Community health assessment and 2 ^nd^ venous blood draw

***Community health assessment.*** Study nurses will visit all mothers or enrolled neonates at their homes at least once. At that visit, a physical examination of the neonate will be completed. The following information will also be collected: neonatal immunisation history (date, time and type), a complete review of systems of the mother and baby, and any medication given to the mother or neonate since delivery. Mothers will also be provided with health education and study contact details (should the neonate become unwell). The effect of vaccination on iron and inflammatory parameters in the first week of life will be analysed as part of the formal data analysis plan.

***2 ^nd^ venous blood draw Group D (FTB+NBW) only.*** At this point, if the mother and neonate are deemed to have passed the screening process and the neonate is in Group D, then the neonate will have its second and last venous blood draw (3.5ml). All samples will reach the laboratory within three hours post collection for primary processing.

### Laboratory evaluations

***Blood samples.*** The processing of samples will be conducted 1-6 hours after collection to allow serum samples to coagulate and to allow mothers admitted via route 2 to give written consent. On arrival to the laboratory, whole blood samples in 4.5ml Sarstedt S-Monovette K3E (SARSTEDT AG & Co. KG, Germany) will be inverted five times and assessed for a full haematology panel using a Medonic M20M GP (Boule Diagnostics, Spanga, Sweden). The remaining whole blood sample will be aliquoted into 2ml Sarstedt micro tubes (SARSTEDT AG & Co. KG, Germany) and frozen at -20°C. Glucose-6-phosphate dehydrogenase deficiency testing (R&D Diagnostics Limited, Papagos, Greece) and sickle trait will be conducted at the end of the recruitment period of the study.

The Sarstedt S-Monovette Serum-Gel blood collection tube (SARSTEDT AG & Co. KG, Germany) will be centrifuged at 3500rpm for 10 mins using an Eppendorf Centrifuge 5702 (Eppendorf, Germany). Serum will be aliquoted into 2ml Sarstedt micro tubes (SARSTEDT AG & Co. KG, Germany), boxed and labelled before being stored at -20°C until after the recruitment period. The laboratory analysis of all serum samples will be conducted as one batch. All serum samples collected will be assessed by ELISA for the following: IL6, IL22, free haem, hepcidin, hemopexin, lipocalin-2, lactoferrin, and foetal haemoglobin. DRG Hepcidin 25 (bioactive) HS ELISA (EIA-5782) (dynamic range: 0.135 - 81 ng/mL), made by DRG Instruments GmbH, Germany, will be used to measure serum hepcidin. Additionally, serum ferritin, serum iron, UIBC, soluble transferrin receptor (sTfR), transferrin, C-reactive protein (CRP), haptoglobin, and alpha 1-acid glycoprotein (AGP) will be assessed using a fully automated biochemistry analyser (Cobas Integra 400 plus). Furthermore, umbilical WBC will be processed and analysed for exploratory secondary analysis 4.

***Bacterial growth assays.*** *Ex vivo* growth of bacteria (including clinical and laboratory isolates of *Staphylococcus aureus, Klebsiella pneumoniae, Escherichia coli, Enterobacter* spp. *, Enterococcus* spp., and *Salmonella* Typhimurium) in a 1:1 mixture of participant serum and Iscove’s Modified Dulbecco’s Medium (IMDM, Invitrogen) as in Cross *et al.* (2015) ^53^ will be performed.

### Study outcomes

The primary outcome variables will be TSAT (transferrin saturation) and serum iron.

The secondary outcome variables will be hepcidin; hemopexin; haptoglobin; IL22; free serum haem and haemoglobin; foetal haemoglobin; lactoferrin; lipocalin-2; IL6; C-reactive protein; alpha-1-acid glycoprotein; transferrin concentration; soluble transferrin receptor; unbound iron-binding capacity; ferritin; haemoglobin; WBCs types and numbers in cord blood samples and *ex vivo* bacterial growth.

### Data entry, handling, storage and security

All protocol-required field data will be captured electronically on an electronic eCRF or a paper case report form (CRF) that will be completed for each included participant. After giving written consent the pregnant women will be given a study identification number, which will be used in all future datasets for subject anonymity. Field data will be collected verbally and from antenatal cards by study nurses. Collected data will be entered in real time using eCRFs developed on top of a REDCap (Research Electronic Data Capture) database and published on Samsung Galaxy Tab 3 SM-T111 handheld devices. Collected data will be transported to the database via a direct secure connection over the 4G mobile network. Laboratory related data will be extracted directly from laboratory equipment and uploaded to the database. Any data collected on the paper format will be double entered by a trained data entry clerk. The local co-investigator will review all forms and identify any errors prior to data entry or to marking data as complete. The study data will also be validated through automated and manual validation methods implemented in the study database application system. The study database will be custom-developed. All paper CRF will be stored in a locked file archive. Electronic data will be stored on the local dedicated server maintained at MRCG. The study will be conducted in compliance with Good Clinical Practice. Study personal security measures will include controlled access limited to authorised users only, physical security, remove identifiable information (anonymization), avoidance of third-party cloud storage and password protection.

### Sample size and power

This study will target recruitment of 150 ‘’exposed” neonates which will include a target of 50 neonates in each Group A, B and C. 300 neonates will be recruited for Group D (“unexposed”). The study will have constraints from time, budget, loss to follow up, haemolysis during sample collection, insufficient blood volume and the distribution of new births in each group at the Kanifing General Hospital. 

Based on this, we have run simulations ( Stata/IC 15.1) to calculate the power to detect a range of differences comparing groups for example Groups D and A with respect to the primary outcomes TSAT and serum iron. We did not calculate power for the secondary outcomes, which are considered exploratory. The simulation was run using a linear regression model assuming a lognormal distribution for the response variables TSAT and serum iron levels 6–24 hours after birth. Data from a previous study (Prentice S, personal communication) was used to obtain mean and SD estimates for TSAT and serum iron both at baseline and 6–24 hours after birth. The predictor variables were the Groups (A–D) with Group D as the reference. The model was adjusted for the baseline (cord blood levels). We also examined the power assuming a normal distribution for TSAT (i.e. without log transformation). The significance level considered was 0.05 and the simulation was run for 100000 iterations. This process was repeated for the following four different sample size scenarios which we refer to as *N1*, *N2*, *N3* and *N4* respectively:

*N1*) Group *A*=Group *B* =Group C =50 neonates
*N2*) Group *A*=Group *B* =25 neonates and Group C =50 neonates
*N3*) Group *A*=Group *B* =10 neonates and Group C =50 neonates
*N4*) Group *A*=Group *B* =50 neonates and Group C =10 neonates

For all the above four cases, *D*=300.

**Figure 3.**
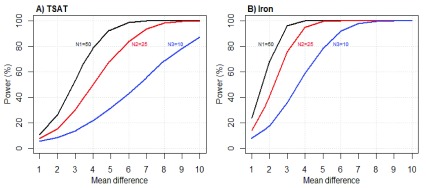
Estimated power to detect a given difference between Groups *A* vs *D* based on simulation using a linear regression model adjusted for baseline for three sample size scenarios. *N1* (Group A=Group B=Group C=50 neonates); *N2* (Group *A*=Group *B* =25 neonates and Group C=50 neonates); *N3* (Group *A*=Group *B* =10 neonates and Group C=50 neonates); *N4* (Group *A*=Group B=50 neonates and Group C =10 neonates).

The simulation results for the baseline adjusted model with log transformation show that for sample size scenario *N1*, the minimum mean differences that can be detected with 80% power were about 4% and 2.5 μmol/L for TSAT and serum iron respectively ( Figure 3A and 3B). These correspond to effect sizes of 0.35 and 0.39 respectively. The power drops substantially if smaller numbers were to be recruited as in scenarios *N2* ( *A*= *B*=25) and *N3* ( *A*= *B*=10). Under *N2* and *N3*, the minimum mean differences that can be detected with 80% would increase to about 5.8% and 9.1% for TSAT ( Figure 3A) and 3.3 μmol/L and 5 μmol/L for serum iron ( Figure 3B). The results for scenario *N4* can be considered as subset of *N1*- *N3* by rearranging Groups A, B and C.

### Statistical analysis

The primary research objective is to examine if preterm and/or low birthweight neonates (“Exposed”) have a reduced ability to sequester iron at 6–24 hours after birth in comparison to full term neonates with normal birthweight (“Unexposed”)?

**Figure 4.**
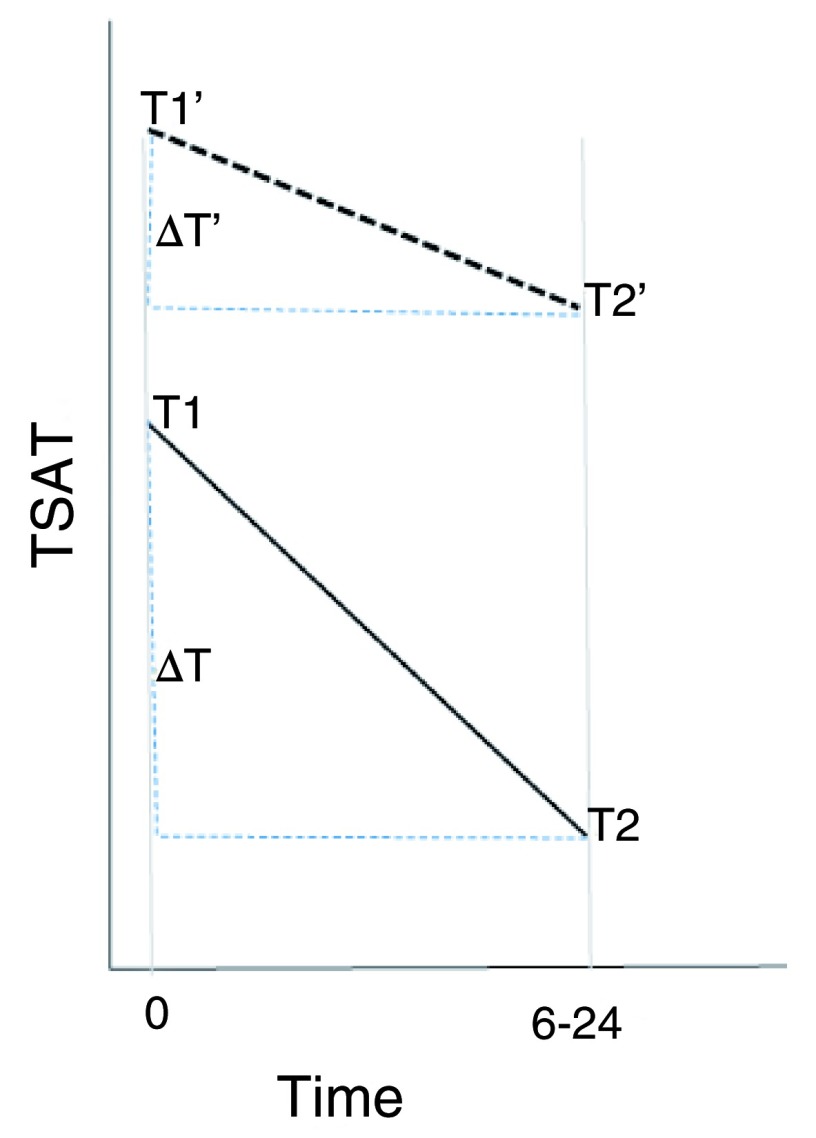
An example of hypothetical scenario for TSAT values between the groups to be compared. In this example: (i) Time 0 refers to average cord blood levels (ii) Time 6–24 refers to the mean level in the 6–24 hour period after birth. (iii) T1, T2, represent TSAT in 1 and 2 above and ΔT=T2-T1 for full term, normal birthweight (Group D) (iv.) T1’, T2’and ΔT’=T2’-T1’ same as above but for the premature, low birthweight (Group A). Hypothesis: H _0_: T2=T2’ vs. H _A_: T2≠T2’.

We hypothesize that FTB+NBW (Group D) neonates on average will have lower values of TSAT and serum iron compared to “Exposed” (PTB or LBW babies) ( Figure 4). Initially, we will analyse all “Exposed” (Groups A+B+C) vs “Unexposed” (Group D). Each neonate will be further classified by his or her gestational age (premature vs. full term) and birthweight (low vs. normal) in a 2x2 table ( Table 1). Linear regression models will be used in order to evaluate the difference in mean between each Group A–C and D; that is where *D* will be the reference group. TSAT and serum iron levels will be log transformed before fitting the models (if necessary). Both the unadjusted and adjusted (for the cord blood level) mean differences together with the 95% CI will be calculated.

**Table 1.** Four combinations in total for exposure: *a*: Pre, Low; *b*: Pre, Normal; *c*: Full, Low and *d*: Full, Normal.

		Birthweight (BW)	
		Low	Normal
Term	**Pre**	A	B
	**Full**	C	D

In the second stage of analysis, we will assess the effect of potential confounding variables using the regression models. Covariates to be considered include the specific time of measurement, demographic and health variables. The time effect may not be linear, and this will be investigated in the further regression models. To reduce the effects of multiple testing, data analysis will be driven by a predefined primary study hypothesis. Any exploratory analyses conducted (in the absence of predefined study hypotheses) will be considered hypothesis-generating, rather than confirmatory. In order to reduce the levels of missing and inaccurately entered data into the database, all clinical, demographic and laboratory data will be entered in real time via electronic data capture, with automated and manual validation methods implemented. The study design does not provide for the recruitment of equal numbers of subjects in each month of the year (or during the dry vs wet seasons). The Gambia has a higher birth rate during the months of September–December in comparison to other months ^54^.

In order to remove this potential source of bias, we will adjust for month of birth and/or season in the regression analysis. If the missing data rates is more than 5%, we will consider imputation. The follow-up duration is relatively short. Thus, we expect little bias from loss to follow-up. If loss to follow-up rate is considerably different between groups, we will perform sensitivity analyses to examine the robustness of results. We will also consider sensitivity analysis fitting a multivariate regression model where the main outcomes of interest (including TSAT, serum iron and hepcidin) will be jointly regressed to the same set of predictors.

The analysis for the secondary objectives are described below:

I. **Characterise how iron metabolism, handling and recycling differs between full term, preterm and low birthweight neonates at birth and during the first 24 hours of life.**

A similar strategy will be used as for the primary objective. Regression modelling will be used to evaluate the difference in means between each Group A–C and D; D will be the reference (“Unexposed”) group. The effects of potential confounding variables will also be assessed using further regression modelling.

II. **Describe iron metabolism, handling and recycling in full term neonates at birth and during the first 7 days of life.**

Analysis of the longitudinal data will involve generalised estimating equations incorporating time of measurement. We will include spline terms at each time point to evaluate the change in the outcomes (all primary and secondary outcome parameters) over time during the transition period from cord to 24–79; 80–135; 136–192 hours after birth. Note that this will only include data collected from Group D neonates and will not be a comparison between Groups A–C and D neonates.

III. **Determine if sera from preterm and low birthweight neonates supports a greater level of *ex-vivo* growth of microorganisms that are common causes of neonatal sepsis in Africa and The Gambia ( *Staphylococcus aureus, Klebsiella pneumoniae, Escherichia coli, Enterobacter* spp. *, Enterococcus* spp., and *S.* Typhimurium) in comparison to sera from FTB+NBW neonates.**

The bacterial growth will be analysed in a similar method as described in Cross *et al.* (2015) ^53^ in order to determine if changes in iron availability modulate the growth. Growth assays will be fitted to a standard form of the logistic equation:

$$N_{t}=\frac{K}{1+(\frac{K-N_{0}}{N_{0}})e^{-rt}}$$

Here, the population size at the beginning of the growth curve is given by *N _0_.* The carrying capacity is given by *K*. The intrinsic growth rate of the population is *r*. We will generate the best fitting values of *K*, *r* and *N _0_* for the growth curve data. Additionally, for each bacterium, we compare the time at which the population density reaches $\frac{1}{2}K$ (inflection point), the fastest possible generation time (doubling time) and the area under the logistic curve obtained by taking the integral of the logistic equation. This will be used to assess growth curves from different sample types (Cord vs V1) and between the four study groups.

**IV. Characterize frequencies and functionality of neutrophils, monocytes, dendritic cells, NK cells, B cells, T cells (D8 and CD4) in cord blood from full term, premature and low birthweight neonates.**

Exploratory analysis will be conducted using linear regressions modelling.

Statistical analyses will be performed using STATA (StataCorp. 2017. Stata Statistical Software: Release 15. College Station, TX: StataCorp LLC); R (R Foundation for Statistical Computing, Vienna, Austria.) and Data Desk (Data Description Inc Ithaca NY). All files used will have an accompanying data dictionary. Annotated STATA do-files or R files will be used to describe any data transformations and statistical tests used.

### Dissemination of findings

The study results will be published in relevant peer-reviewed journals and key findings will be presented at international scientific meetings. Data sharing will be in agreement with the sponsor policy on research data sharing and with the Bill & Melinda Gates Foundation Global Access requirements.

### Study status

The study is in the data collection phase.

## Discussion

Humans and bacteria are involved in an on-going tug of war over iron. Each side has evolved complicated and varied iron-acquisition mechanisms in an effort to turn the tide of war in their own favour ^55^. Nutritional immunity describes the processes by which the human host tries to starve invading organisms of nutrients, especially iron.

This study aims to determine if premature and low birthweight babies have a defect in their ability to sequester iron during the first 24 hours of life. The study design will produce a detailed and extensive picture of iron metabolism in neonates. To our knowledge, no other study has tried to analyse such a large and diverse collection of iron and infection variables in neonates born in Sub Saharan Africa. The study will enrol subjects who are all at an increased risk of neonatal infection, and subsequent sepsis and death.

A potential limitation of this study is the inadequacy of using the New Ballard Score as the only method of gestational aging. Original and New Ballard Score are reported to overestimated gestational age compared to ultrasound and in particular, misclassify preterm infants as term newborns ^56^. Additionally, newborn clinical assessments as a whole, tend to underestimate gestational age in growth-restricted neonates ^56^. The gold standard of gestational aging is an ultrasound in the first trimester ^57^. However, this procedure is rarely correctly completed in this study population. If it is documented on the mother’s antenatal records, care will be taken to record it. Limits of the study also include that HIV status, TB status and iron supplementation given are all gained from the antenatal records of the mother. Furthermore, antenatal records will not contain all information on medication given in every mothers’ pregnancy. As a result, care will be made to extensively question participants mother’s during verbal one-to-one consultation with our study research clinician.

In conclusion, our overarching study goal is to evaluate the likelihood that novel products designed to induce hypoferremia (potentially via mini-hepcidins ^58^) may be useful in the future for the prevention of neonatal sepsis in high risk babies. This could be produced by a transient redistribution of iron away from the circulation, thus applying a bacteriostatic brake on any bacteria that have crossed into the baby’s systemic circulation and hence boosting host survival in vulnerable newborns. We hope this may ultimately help reduce the use of antibiotics in maternal and neonatal wards worldwide.

## Ethical approval

This study has been approved by The Gambia Government/MRC Joint Ethics Committee (no. SCC1525) and Ethics Committee of London School of Hygiene and Tropical Medicine (ref no. 14316). The study procedures will be explained to the neonate’s mother/guardians orally or in writing. A neonate is only recruited into the study after the consent form has been signed/thumb printed by the mother/guardian.

This study was registered with clinicaltrials.gov ( NCT03353051) on 27 November 2017.

## Data availability

### Underlying data

No data are associated with this article.

### Extended data

Figshare: Cross *et al.* GatesOpen Research SCC1525v2__NeoInnate_Participant Info&Consent form Route 1. https://doi.org/10.6084/m9.figshare.8069195.v4 ^49^

This project contains the following extended data:

- SCC1525v2__NeoInnate_Consent form Route 1_v3 Approved8Nov17.docx (Route 1 consent and information sheet)

Figshare: Cross *et al.* GatesOpenResearch SCC1525v2_NeoInnate_Consent form Route 2_Part 1_ (Umbilical Cord Blood Collection) - Labour Ward_v1.1-Approved 8Nov17. https://doi.org/10.6084/m9.figshare.8069246.v1 ^50^

This project contains the following extended data:

- SCC1525v2_NeoInnate_Consent form Route 2_Part 1_ (UCB Collection) - Labour Ward_v1.1-Approved 8Nov17.docx (Route 2 consent and information sheet part 1)

Figshare: Cross *et al.* Gates Open Research SCC1525v2__NeoInnate_Consent form Route 2_Part 2_(Post-Delivery) - ANC Outside SGH v1-Approved 8Nov17. https://doi.org/10.6084/m9.figshare.8069243.v1 ^51^

This project contains the following extended data:

- SCC1525v2__NeoInnate_Consent form Route 2_Part 2_(Post-Delivery) - ANC Outside SGH v1-Approved 8Nov17.docx (Route 2 consent and information sheet part 2)

Data are available under the terms of the Creative Commons Attribution 4.0 International license (CC-BY 4.0).
