# Supplementary material for: Neonatal iron distribution and infection susceptibility in full term, preterm and low birthweight babies in urban Gambia: study protocol for an observational study
Source: Gates Open Res. 2019 Oct 15;3:1469. Originally published 2019 May 21. [Version 2] doi: 10.12688/gatesopenres.12963.2 (PMC6757319; doi:10.12688/gatesopenres.12963.2)
Supplement: Peer review history

# Peer review: Reviewer response for version 1

**DOI:** 10.21956/gatesopenres.14069.r27774

**Published:** 2019-09-19

**Authors:** Veena Sangkhae

**Affiliations:** 1 Center for Iron Disorders, David Geffen School of Medicine, University of California, Los Angeles (UCLA), Los Angeles, CA, USA

The observational study proposed by Cross *et al *aims to study the role of neonatal iron metabolism on the susceptibility of neonates to infection. Subjects will be recruited in Serrekunda and deliver at Kanifing General Hospital in The Gambia, West Africa. The study will include 4 groups: 1) full-term normal birthweight, 2) full-term low birthweight, 3) preterm normal birthweight and 4) preterm low birthweight neonates. At delivery, blood will be collected from the umbilical arteries and vein. In addition, neonatal venous blood will be collected between 6 and 24hrs following delivery. A full hematology panel will be performed on whole blood and various inflammatory markers and iron status indicators will be measured in serum. The primary objective of the study is to determine how full term, preterm and low birthweight neonates regulate their serum iron in the 24 hours after birth. Cross *et al *hypothesize that preterm and/or low birthweight neonates will be unable to sufficiently regulate serum iron levels and transferrin saturation, thus making them more susceptible to infection.

 The rationale for, and objectives of the study are clearly described. Neonatal infection remains a major global health concern and gaining a detailed understanding of iron metabolism in neonates and their susceptibility to infection may facilitate better monitoring practices, development of improved diagnostics and novel therapies.

 The study design is appropriate for the research question. Measurement of neonatal serum iron concentration and transferrin saturation will provide information on the bioavailability of iron at baseline for microorganisms. Determining *ex vivo* growth of microorganisms in sera from term and preterm neonates will provide additional information on the susceptibility of neonates to infection. However, inclusion of neonates that have received immunizations prior to the 1 ^st^ venous blood draw at 6-24 post-delivery may introduce a confounding factor. In adults, 24hrs following immunization, both IL-6 and hepcidin are significantly increased (Stoffel *et al. *2019 ^1^). Thus, for the 2 ^nd^ venous blood draw of the full-term normal birthweight group, it may be useful to delay the blood draw if a neonate has received an immunization within the previous 24hrs. An additional point that needs some clarification is exclusion criteria delta which lists cord blood processed >3hrs. However, for route 2 enrollment, cord blood will be collected and stored until consent is obtained which is listed at 2-6hrs following delivery, which could mean a large number of route 2 enrollment samples are excluded, could you please provide clarification.

 The methods provided for the most part allow for replication by others but some of the details are not included. The manner of blood collection (whole blood in EDTA, serum), time ranges and storage conditions are listed; however, details regarding processing and storage are missing. Will all samples be processed for all of the parameters immediately after collection or will parameters in whole blood processed immediately but serum collected and stored at -20C and processed together following collection of all samples? The panel of cytokines and iron status indicators to be measured are listed; however, for samples analyzed by ELISA, it would be informative to list the specifics of each ELISA (i.e. manufacturer).

 Authors sufficiently indicate the limitations of the study. However, despite these limits, the data gathered will provide valuable insight into iron regulation during this period of human development. This study is of great interest as neonates are at greater risk of infection due to their underdeveloped immune systems, yet nutritional immunity including neonatal iron regulation and distribution is not well characterized. Although the iron-infection axis has been extensively studied in the adult population, it is understudied in neonates. This is specifically of interest in regions where are neonatal infections are common. Vaginal birth, a requirement in this study, is associated with increased inflammation, which induces hepcidin in the mother and has been postulated to increase hepcidin in the fetus. This is a confounding factor in some studies as conclusions regarding hepcidin and iron regulation at delivery are made using cord blood which likely has elevated hepcidin due to inflammation associated with delivery. It is unique and important that this study will provide information from neonatal blood at >24 to <80h; >80h to <136; and >136h to <192h after delivery. The proposal to measure both cord blood in addition to neonatal blood will inform on whether cord blood is an accurate indicator of neonatal iron status. Not only will this study provide an increased understanding of nutritional immunity in neonates, it will also provide much needed information on iron homeostasis during the neonatal period.

I confirm that I have read this submission and believe that I have an appropriate level of expertise to confirm that it is of an acceptable scientific standard.

## References

[1] : The opposing effects of acute inflammation and iron deficiency anemia on serum hepcidin and iron absorption in young women. *Haematologica*.2019;104(6) : 10.3324/haematol.2018.208645 1143-1149 10.3324/haematol.2018.208645 30630976PMC6545852

# Response

**Published:** 2019-10-02

**Authors:** Carla Cerami

**Query:**  The study design is appropriate for the research question. Measurement of neonatal serum iron concentration and transferrin saturation will provide information on the bioavailability of iron at baseline for microorganisms. Determining *ex vivo* growth of microorganisms in sera from term and preterm neonates will provide additional information on the susceptibility of neonates to infection. 

 However, inclusion of neonates that have received immunizations prior to the 1 ^st^ venous blood draw at 6-24 post-delivery may introduce a confounding factor. In adults, 24hrs following immunization, both IL-6 and hepcidin are significantly increased (Stoffel *et al. *2019 ^1^). Thus, for the 2 ^nd^venous blood draw of the full-term normal birthweight group, it may be useful to delay the blood draw if a neonate has received an immunization within the previous 24hrs. 

**Response:**

**Please note that this same question has also been raised by Dr Stefanova.**

 According to current practice, the vaccination of neonates at Kanifing General Hospital (The Gambia), occurs at a later time point due to social and logistical reasons. However, the mothers will be asked whether the neonate has been vaccinated at the health check before the 1 ^st^venous bleed takes place. The answers will be recorded and this data will be included in the final report.

 The embedded observational study (which includes only full- term babies) includes a home visit up to seven days after birth for a health check and collection of a 2 ^nd^venous blood sample. At this visit data will also be collected on the type, time and date of vaccination. The effect of vaccination on iron and inflammatory parameters in the first week of life will be analysed as part of the formal data analysis plan. 

**Query:**

 An additional point that needs some clarification is exclusion criteria delta which lists cord blood processed >3hrs. However, for route 2 enrolment, cord blood will be collected and stored until consent is obtained which is listed at 2-6hrs following delivery, which could mean a large number of route 2 enrolment samples are excluded, could you please provide clarification.

**Response:**

 To clarify, mothers will be approached 2-6 hours following delivery. Samples will still be processed up until 6 hours post-collection. This information will be recorded and assessed as part of the formal data analysis plan. Any values that are greater or lesser than 5 standard deviations of the mean will be regarded as outliers and removed. 

**Query:**

 The methods provided for the most part allow for replication by others but some of the details are not included. The manner of blood collection (whole blood in EDTA, serum), time ranges and storage conditions are listed; however, details regarding processing and storage are missing. Will all samples be processed for all of the parameters immediately after collection or will parameters in whole blood processed immediately but serum collected and stored at -20C and processed together following collection of all samples? 

**Response:**

 The processing of samples will be conducted 1-3 hours after collection to allow serum samples to coagulate and to allow mothers admitted via route 2 to give written consent. Whole blood in EDTA will be run using the Medonic M20M GP (Boule Diagnostics, Spanga, Sweden) after inversion of the blood collection tube 5 times. Remaining whole blood samples will be aliquoted into 2ml Sarstedt micro tubes (SARSTEDT AG & Co. KG, Germany) and frozen at -20°C. G6PD deficiency testing (R&D Diagnostics Limited, Papagos, Greece) will be conducted at the end of the recruitment period in the study. 

 The Serum-Gel blood collection tube (SARSTEDT AG & Co. KG, Germany) will be centrifuged at 3500rpm for 10 mins using an Eppendorf Centrifuge 5702 (Eppendorf, Germany). Serum will be aliquoted into 2ml Sarstedt micro tubes (SARSTEDT AG & Co. KG, Germany), boxed and labelled before being stored at -20°C until after the recruitment period.  All laboratory analysis of iron and inflammatory parameters will be conducted as one batch. 

 The panel of cytokines and iron status indicators to be measured are listed; however, for samples analyzed by ELISA, it would be informative to list the specifics of each ELISA (i.e. manufacturer).

**Response:**

 DRG Hepcidin 25 (bioactive) HS ELISA (EIA-5782) (dynamic range: 0.135 - 81 ng/mL), made by DRG Instruments GmbH, Germany, will be used to measure serum hepcidin. The results of the DRG ELISA (ng/nl hepcidin) will be presented along with the standardised hepcidin results as described in Van der Vorm et al ^1^.  

 1.        van der Vorm, L. N. *et al.*Toward Worldwide Hepcidin Assay Harmonization: Identification of a Commutable Secondary Reference Material. *Clin. Chem.* **62**, 993–1001 (2016).

# Peer review: Reviewer response for version 1

**DOI:** 10.21956/gatesopenres.14069.r27647

**Published:** 2019-09-03

**Authors:** Deborah Stefanova

**Affiliations:** 1 Institute for Molecular Health Sciences, ETH Zurich, Zurich, Switzerland

The study by Cross *et al.* proposes to investigate whether regulation of iron metabolism during the first 24h of life differs between full-term normal birth weight newborns and newborns that are either low birth weight, pre-term or both. For this purpose, blood will be collected for analysis from the umbilical cord and compared to venous blood collected from the newborns at 6 to 24h post-delivery. In addition, an observational study to gain insight into neonatal iron metabolism during the first 8 days of life will be conducted within the full-term normal birth weight newborns group. The obtained blood will be analyzed for various iron parameters, cytokines, full haematology count etc. The authors hypothesize that newborns that are either low birth weight, pre-term or both will be unable to efficiently decrease transferrin saturation (TSAT) and serum iron and as a result they will become more susceptible to infections. In order to partially address this hypothesis, different bacteria that commonly infect newborns in the region where the study is conducted, will be cultured in plasma obtained from each group of newborns and bacterial growth will be compared.

 The rationale and the objectives of the study are clearly defined. The study design appropriately addresses the research question and offers one of the first studies where blood from newborns will be analyzed at 6h to 192h post-delivery. Many studies so far have focused predominantly on umbilical cord blood, which while informative, can also be quite misleading since the stress of birth has significant effect on hepcidin production and thus on iron parameters. The methods are described clearly and in detail.

 It would be good if at Route 2 of study enrolment, where the mothers will be invited to provide written consent at 2-6h post-delivery, a brief assessment of the psychological state of the mother is done beforehand since 2-6h is still quite early after giving birth. In addition, immunizations are not an exclusion criteria for newborn blood collection in this study, which makes sense since most newborns receive immunizations within the first 24h of their life. However, activating the immune response has an effect of hepcidin production so a note should be taken on whether or not a newborn has been immunized before blood collection for future reference. Lastly, the cited method for growing bacteria in human plasma uses 50% human plasma but it is not clear what composes the other 50% of the culture medium (whether it is regular commercial growth medium such as Luria-Bertani or some kind of buffer). It is important to define that, since commercial growth medium contains small but sufficient amount of free iron, which is readily available for bacterial acquisition and could affect bacterial growth. If possible, the bacteria could be cultured in 90-100% human plasma to avoid such confounding factors.

 The study by Cross *et al.* addresses a very interesting and important biological question regarding the regulation of iron metabolism in neonates and its relevance for susceptibility to infection. I expect that the information gathered through this study will contribute significantly to the knowledge in the community.

I confirm that I have read this submission and believe that I have an appropriate level of expertise to confirm that it is of an acceptable scientific standard.

# Response

**Published:** 2019-10-02

**Authors:** Carla Cerami

**Query:**

 It would be good if at Route 2 of study enrolment, where the mothers will be invited to provide written consent at 2-6h post-delivery, a brief assessment of the psychological state of the mother is done beforehand since 2-6h is still quite early after giving birth. 

**Response:**

 Mothers will be approached 2-6 hours post-delivery on a one-to-one basis by our research clinician. The research clinician will independently verify if the mother displays normal psychological function and is not under distress before the consenting process begins. Good clinical practise will be followed at all times. 

**Query:**

 In addition, immunizations are not an exclusion criteria for newborn blood collection in this study, which makes sense since most newborns receive immunizations within the first 24h of their life. However, activating the immune response has an effect of hepcidin production so a note should be taken on whether or not a newborn has been immunized before blood collection for future reference. 

**Response:**

 According to current practice, the vaccination of neonates at Kanifing General Hospital (The Gambia), occurs at a later time point due to social and logistical reasons. However, the mothers will be asked whether the neonate has been vaccinated at the health check before the 1 ^st^venous bleed takes place. The answers will be recorded and this data will be included in the final report.

 The embedded observational study (which includes only full- term babies) includes a home visit up to seven days after birth for a health check and collection of a 2 ^nd^venous blood sample. At this visit data will also be collected on the type, time and date of vaccination. The effect of vaccination on iron and inflammatory parameters in the first week of life will be analysed as part of the formal data analysis plan. 

**Query:**

 Lastly, the cited method for growing bacteria in human plasma uses 50% human plasma but it is not clear what composes the other 50% of the culture medium (whether it is regular commercial growth medium such as Luria-Bertani or some kind of buffer). It is important to define that, since commercial growth medium contains small but sufficient amount of free iron, which is readily available for bacterial acquisition and could affect bacterial growth. If possible, the bacteria could be cultured in 90-100% human plasma to avoid such confounding factors.

**Response:**

 The culture medium used will be iron free minimal growth media, Iscove’s Modified Dulbecco’s Medium (IMDM, Invitrogen). The *ex vivo*bacterial growth assay has been previously conducted in pilot experiments, using 90-100% human serum, however, growth rates diminished to a level that was difficult to detect.
